# Prevention of *Clostridium difficile* Infection and Associated Diarrhea: An Unsolved Problem

**DOI:** 10.3390/microorganisms8111640

**Published:** 2020-10-23

**Authors:** Nicola Principi, Margherita Gnocchi, Martina Gagliardi, Alberto Argentiero, Cosimo Neglia, Susanna Esposito

**Affiliations:** 1 Università degli Studi di Milano, 20122 Milan, Italy; nicola.principi@unimi.it; 2 Pediatric Clinic, Pietro Barilla Children’s Hospital, University of Parma, 43126 Parma, Italy; margherita.gnocchi@gmail.com (M.G.); marty.gagliardi@hotmail.it (M.G.); alberto.argentiero@unipr.it (A.A.); cosimo.neglia@unipr.it (C.N.)

**Keywords:** *Clostridium difficile*, *Clostridium difficile*-associated disease, *Clostridium difficile* infection, diarrhea, vaccines

## Abstract

For many years, it has been known that *Clostridium difficile* (CD) is the primary cause of health-care-associated infectious diarrhea, afflicting approximately 1% of hospitalized patients. CD may be simply carried or lead to a mild disease, but in a relevant number of patients, it can cause a very severe, potentially fatal, disease. In this narrative review, the present possibilities of CD infection (CDI) prevention will be discussed. Interventions usually recommended for infection control and prevention can be effective in reducing CDI incidence. However, in order to overcome limitations of these measures and reduce the risk of new CDI episodes, novel strategies have been developed. As most of the cases of CDI follow antibiotic use, attempts to rationalize antibiotic prescriptions have been implemented. Moreover, to reconstitute normal gut microbiota composition and suppress CD colonization in patients given antimicrobial drugs, administration of probiotics has been suggested. Finally, active and passive immunization has been studied. Vaccines containing inactivated CD toxins or components of CD spores have been studied. Passive immunization with monoclonal antibodies against CD toxins or the administration of hyperimmune whey derived from colostrum or breast milk from immunized cows has been tried. However, most advanced methods have significant limitations as they cannot prevent colonization and development of primary CDI. Only the availability of vaccines able to face these problems can allow a resolutive approach to the total burden due to this pathogen.

## 1. Introduction

*Clostridium difficile* (CD) is a toxin-producing, Gram-positive, spore-forming, anaerobic pathogen that can simply colonize the intestinal tract or be associated with gastrointestinal manifestations of various severity from mild to life threatening that can frequently recur. Severe cases, such as those with pseudomembranous colitis, toxic megacolon, perforation of the colon, and septic shock are diagnosed in about 1% of hospitalized patients and death can occur in up to 17% of them, with the highest values during outbreaks [1,2]. According to the European Center for Disease Prevention and Control, CD infection (CDI) is diagnosed when a patient presents with diarrheal stools or toxic megacolon and a positive laboratory assay for CD toxin A and/or B in stools or a toxin-producing CD organism detected in stool via culture or other means e.g., a positive PCR result; or pseudomembranous colitis revealed by lower gastro-intestinal endoscopy; or colonic histopathology characteristic of CDI (with or without diarrhea) on a specimen obtained during endoscopy, colectomy, or autopsy [3]. For many years, it has been known that CD is the primary cause of health-care-associated infectious diarrhea, afflicting many hospitalized patients [1]. However, recent studies have shown that CD can also play a relevant role as a cause of disease in the community, as more than 50% of CDIs have an onset in the community [4]. A study carried out in the USA estimated that in 2011, CD caused just under half a million infections, with approximately 80,000 recurrences and approximately 30,000 deaths [5]. In the EU/EEA, the burden of healthcare-associated CDIs in acute care hospitals it has been estimated at 123,997 cases annually with a number of deaths of about at 3700 [6]. Even higher yearly prevalence of CDI was reported in other studies [7,8] or was calculated by means of mathematical models [9]. In general, studies indicate that both the incidence and severity of CDI have tended to increase, mainly because of the emergence of the highly virulent CD BI/NAP1/027 strain [10]. In the USA, hospital stay for CDI quadrupled between 1993 and 2009 [9], and mortality doubled between 1993 and 2003 [11]. In Europe, in 2008, hospital incidence of CDI was found to be 4.1 cases per 10,000 patient-days, a value almost 70% higher than that reported in a previous European surveillance study in 2005 (2.45 cases per 10,000 patient-days) [12]. 

Prolonged antibiotic use is the main risk factor for CDI. Other favoring factors are prolonged hospital stay, age >65 years, immunosuppression, the presence of severe underlying illness, and the use of antiulcer and chemotherapeutic medications [13,14]. Together with medical problems, CD causes substantial economic costs. In a study enrolling a total of 55,504 CDI patients, among whom approximately 25% had a recurrent episode, it was found that the mean number of hospitalization days was 5.20 days and 1.95 days for primary CD infections and recurrences, respectively. Costs were USD 24,205 for primary infections and USD 10,580 for recurrences [15].

To reduce the total CD burden, infection prevention has been repeatedly recommended, and several clinical practice guidelines have been developed [16]. Antibiotic stewardship, compliance with CDC, WHO and the European Society of Clinical Microbiology and Infectious Diseases hand-hygiene and contact precaution recommendations, and the use of a sporicidal disinfectant or diluted sodium hypochlorite for environmental cleaning and disinfection are the most commonly recommended strategies to prevent CD infection development and diffusion [17,18]. Moreover, although no guidelines have included them among suggested methods to prevent CD infection and disease, probiotics have been considered by some authors a potential measure in this regard [19]. Finally, attempts to use fecal transplantation [20] and to develop vaccines have been made [21]. In this narrative review, the present possibilities of CDI prevention will be discussed. Studies published in English from January 1990 to May 2020 and indexed in PubMed and Scopus were used. Selection was made using the following key words: CD, CDI, CD diarrhea, CD prevention, CD vaccines, probiotics, fecal microbiota transplantation, antibiotic stewardship, and infection control measures.

## 2. Infection Control Measures

As with other bacteria, the skin and environment of a CD-colonized or infected subject become quickly contaminated and are the basis for CDI diffusion among other patients and healthcare professionals. To reduce this risk, all the guidelines for CDI prevention recommend that general infection control measures be systematically applied where potential patients are located [17,18]. They include: (1) prompt identification of patients with suspected CDI, (2) the isolation of suspected and documented cases, (3) the disinfection of the patient’s room and objects, (4) the use of contact isolation measures such as gloves and gowns, and (5) hand washing.

Early diagnosis of CD carriage or CDI could prevent the spread of the pathogen and the development of recurrences. However, the optimum method of laboratory diagnosis remains controversial. In some cases, a two-step approach beginning with enzyme immunoassays for the detection of glutamate dehydrogenase (GDH) followed by a toxin test and/or a nucleic acid test is recommended. In other cases, a nucleic acid test alone is considered adequate [19]. It is recommended that only patients with unexplained and new onset ≥ 3 unformed stools in 24 h must be tested unless an ileus is present. Furthermore, it is highlighted that testing must not be repeated during the same episode of diarrhea. Children in the first year of life are excluded due to the high prevalence of asymptomatic carriage in these subjects [17,18]. However, whereas the recommendation for children is strong and based on moderate-quality evidence, the one for adults is weak and based on low-quality evidence [16,19]. Patient isolation is generally recommended as is environmental cleaning. The sharing of a room with a patient with CDI has been found to be a risk factor for CDI development [22]. Moreover, environmental cleaning with disinfectants is effective in decreasing patients’ risks of developing CDI only in areas where CDI is highly endemic [23,24]. The use of unbuffered 1:10 hypochlorite solution to clean the rooms of patients with CDIAD was found to reduce the incidence rate of the disease for bone marrow transplant patients who previously had an incidence of 8.6 cases per 1000 patient-days to 3.3 cases per 1000 patient-days. In contrast, CDI rates were not reduced among neurosurgical intensive care unit and general medicine patients, for whom rates before intervention were 3.0 and 1.3 cases per 1000 patient-days, respectively [25].

Hand washing with alcohol-based hand rubs or soap and water has been demonstrated to be very effective for the prevention of the transmission of several infectious diseases, including those due to *Staphylococcus aureus* and vancomycin-resistant *Enterococcus* [26,27,28,29]. Asymptomatic carriage of CD is common in hospitalized patients, and skin colonization with CD can be found in a great number of subjects up to 7 days after the resolution of CDI episodes. The risk of CDI and recurrence in hospitalized patients is extremely high [30]. Consequently, hand washing is one of the measures that are strongly recommended by clinical practice guidelines for the prevention of CDI and recurrence [14]. Unfortunately, CD is a pathogen with peculiar characteristics. It forms spores that are resistant to alcohol-based preparations [31], is poorly removed from contaminated hands by alcohol-based hand rubs [32] and can become the reason for CD transmission. This explains why the use of alcohol-based hand rubs has been debated. However, data collected in clinical trials specifically planned to evaluate the true efficacy of these products are reassuring, as an increase in CDI has never been reported [27,33,34,35,36]. The evidence that in patients with CDI, most of the CD that is recovered in the feces is in vegetative forms and only 10% as spores can explain this finding [36]. Vegetable forms of CD are highly sensitive to alcohol and can be eliminated when exposed to alcohol-containing preparations to effectively reduce the risk of transmission. According to the Australasian College of Infection Prevention and Control, it could be suggested that hands should be washed with soap and water or an antiseptic wash when they are visibly dirty/soiled, and hands should be disinfected with alcohol-based hand rub when they are visibly clean [37]. Moreover, healthcare workers must use gloves on entry to a room of a patient with CDI and while caring for patients with CDI. However, after removing gloves hand washing is mandatory [38]. 

Regardless of the limitations of each of the measures for infection control, the prevention of CDI can be strongly influenced by healthcare worker compliance with contact isolation precautions. Several studies have clearly shown that compliance is generally poor [39]. Adherence to all the recommended infection control measures is a complex, time-consuming process. Moreover, interventions are frequently not precisely defined, and recommendations are ambiguous [40]. In addition, a role in favoring poor adherence can be played by poor supply adequacy of personal protective equipment and hand-hygiene products. In a study in which compliance with all components of contact isolation precautions of health care workers of two hospitals was assessed, it was found that where compliance was poor, at least one supply item for contact isolation precautions was inadequate in most of the studied cases [41].

## 3. Antibiotic Stewardship

Antibiotic stewardship (AS), i.e., the implementation of programmes for the optimization of antibiotic use, is considered one of the most important measures to reduce the selection of host microbiota and the emergence of antimicrobial-resistant bacteria. Moreover, AS has been found to be associated with a significant reduction in antibiotic consumption, related adverse events, and costs for assistance in both hospital and community settings [42]. Use of almost all the antibiotics can increase risk of CDI development, although cephalosporins, fluoroquinolones, clindamycin, and some penicillins, such as co-amoxiclav, are those more frequently associated with these conditions [43]. Most of CDI cases, both in the hospital and in the community, have received antibiotics in the weeks before the diagnosis [42]. Starting from the evidence that antibiotic administration was the most important cause of CDI, it is not surprising that several attempts to rationalize antibiotic use through auditing, antibiotic restriction, antibiotic cycling or mixing, and the reduction of treatment duration to prevent CD colonization and infection have been carried out, mainly in the elderly population [43,44,45,46,47,48,49,50,51,52,53,54,55,56,57,58,59,60,61,62,63,64,65]. The results were generally very satisfactory, as most of these studies showed that the implementation of AS programmes was associated with a significant reduction in CDI incidence after antibiotic use. However, a number of studies, particularly quasi-experimental studies, had numerous potential biases [66], leading to debatable conclusions. In addition, in some cases, they were carried out during outbreaks [67], and the results do not represent what likely occurs in normal periods. Finally, studies were very heterogeneous [16]. Populations enrolled differed in demographic characteristics; underlying disease for which antibiotics were prescribed; the type, dosage, and schedule of drug administration; methods of CDI identification. AS interventions were frequently different and, in some cases, associated with one or more infection control measures. Consequently, although the use of AS interventions remains, together with infection control measures, the most effective available measure to prevent CDI infection after antibiotic therapy, which subjects have the greatest benefit from AS interventions is not precisely defined. Only a few studies included outpatients [16]; children were not considered; frequently, poor importance was ascribed to the underlying disease that justifies antibiotic prescription. It is not clarified which kind of AS interventions can be associated with the highest reduction of CDI risk; how long AS must be maintained; finally, which infection control measures can significantly contribute to limiting CDI development. Limitations of current knowledge on the role of AS in CDI prevention are clearly highlighted by the results of some systematic reviews and meta-analyses carried out using the most important studies in this regard. In a meta-analysis of 16 articles published before 2012 in which quasi-experimental or observational study designs were used and only hospitalized adults were included, AS was associated with a significant protective effect, as the risk of CDI development was reduced by more than 50% (relative risk (RR) 0.48; 95% confidence interval (CI) 0.38–0.62). Complete removal of antibiotics or use allowed only after approval were the most effective types of interventions. However, only nine studies were considered of high quality, most of the data were collected in geriatric wards, the restriction of only fluoroquinolones and cephalosporins was analyzed, and AS duration varied from ≤9 to ≥16 months [68]. Slightly different results were evidenced by a more recent systematic review and meta-analysis [69] based on 11 studies performed up until 2016 that included only hospitalized patients. This study concluded a significant but lower benefit of AS than that found in the previous meta-analysis, as the reduction of CDI infection was only 32% (odds ratio (OR) OR 0.68; 95% CI 0.53–0.88; *p* = 0.0029). Different characteristics of the papers not included in the previous study and different methodologies used for analysis may explain this difference. The impact of the association of AS programmes with infection control measures was analyzed considering together the prevention of CD infection and the prevention of infection and colonization with antibiotic-resistant bacteria. This revealed that the association was more effective than AS alone (IR 0.69, 0.54–0.88; *p* = 0.0030), especially when AS was associated with hand-hygiene interventions (0.34, 0.21–0.54; *p* < 0.0001).

## 4. Probiotics

As antibiotic-related gut dysbiosis with the emergence of CD is the most important cause of CDI [13], it was supposed that the administration of probiotics, i.e., bacteria that confer a health benefit when ingested [70], could reconstitute normal gut microbiota composition and suppress CD colonization in patients given antimicrobial drugs [70]. The reliability of this supposition was measured by several studies. The effectiveness of probiotics was measured by comparing the incidence of CDI after antibiotic administration in patients given probiotics with that found in controls receiving placebo or no treatment. Unfortunately, most of the studies had several methodological limitations. Only in a few cases were randomized controlled trials (RCTs) performed. Moreover, studies were very heterogeneous. Patients with various antibiotic treatments given for different clinical conditions and with different risks of CDI development were enrolled. Inpatients and outpatients were simultaneously considered. Most of the studies were performed in older people. Various diarrhea definitions were used. Several probiotic preparations prescribed in different dosages for various periods of time were administered. Follow-up modalities and duration varied significantly. This explains why systematic reviews and meta-analyses of these studies have led to conflicting results or were unable to draw firm conclusions on the real efficacy of probiotics and how probiotic prophylaxis should be conducted. Only the safety of prophylaxis was clearly established [71,72,73,74,75,76,77,78,79,80,81,82]. Practically, regarding the prevention of CDI, whereas a positive effect was found in the reviews by Shen et al. [74] and Pattani et al. [80], no impact was reported in the meta-analysis by Goldenberg et al. [71] or in two RCTs [72,73]. For the prevention of CDI, probiotics were effective in two studies [71,77] but were not different from placebo or no treatment in three studies [75,78,79].

A good example of the limited knowledge presently available regarding the prophylactic use of probiotics is given by some recent, well-conducted meta-analyses. The first of them is a Cochrane Review including 31 trials published up until March 2017 and 8672 individuals of different ages [71]. Only 10 studies were rated as having a low risk of bias, whereas 21 were considered to have a high or unclear risk of bias. Compared to controls, patients receiving probiotics were found to have a significantly lower risk of developing CDI both when all the studies were considered together and when only studies with a low risk of bias were analyzed. In both cases, CDI was reduced by 60%. CDI occurred in approximately 1.5% of treated patients and in 4% of controls (RR 0.40, 95% CI 0.30–0.52). However, the clinical relevance of probiotics was relatively low, as it was calculated that to have an additional beneficial outcome, 42 patients had to be treated to prevent one case of CDI. A subgroup analysis revealed that no advantage from probiotics could be demonstrated in patients with a low risk of CDI development, whereas the benefit was increased to 70% in patients with a baseline risk >5% (incidence of CDI 11.6% vs. 3.1%, RR 0.30, 95% CI 0.21–0.42). However, contrary to what was evidenced for CDI prevention, probiotics did not influence CDI, i.e., the detection of CD in the stool. Pathogen detection was 15.5% in the probiotics group compared to 17.0% in the control group (RR 0.86, 95% CI 0.67–1.10). Despite several thousand enrolled patients, the importance of probiotic dose and type in conditioning the development of CDAD could not be evaluated. The authors were unable to identify a credible dose-dependent or type-dependent effect, although in a study, it was found that the risk of CDIAD was lower with *Lactobacillus acidophilus* + *Lactobacillus casei* than with *Lactobacillus rhamnosus* (RR 0.21 vs. RR 0.63, *p* = 0.03). Finally, probiotics were safe, well tolerated, and capable of reducing the risk of adverse events associated with antibiotic administration by 17% (RR 0.83, 95% CI 0.71–0.97).

A more recent meta-analysis of randomized controlled trials published up until April 2016 including 6851 participants and most of the studies already considered in the previous meta-analysis was mainly directed to evaluate the impact of probiotics on the risk of CDI after antibiotic administration [83]. Most of the patients were adults, and in a non-marginal number of cases, risks of bias were evidenced. In this study, probiotic administration was associated with reduced CDI odds in both the unadjusted (OR 0.37 95% CI 0.25–0.55) and the adjusted analysis (OR 0.35, 95% CI, 0.23–0.55). Beneficial effects were more evident when two or more antibiotics were used (OR 2.20, 95% CI 1.11–4.37). In contrast, no influence of age, sex, hospitalization, or the use of antibiotics at high risk of CDI was demonstrated. Prophylaxis with multispecies probiotics was more effective than no prophylaxis in reducing the risk of CDI (OR, 0.33; 95% CI, 0.20–0.56; *p* < 0.0001). However, compared to single-species probiotics, prophylaxis with multispecies probiotics was only slightly more effective, with a difference that did not reach statistical significance (OR, 0.41, 95% CI, 0.17–1.00; *p* = 0.051). Moreover, the effect was more evident when prophylaxis was prescribed in settings with a risk of CDI ≥ 5%. As several different preparations were used, differences in protection offered by the various probiotics could not be evaluated. However, comparison between *Saccharomyces boulardii*, the most frequently administered probiotic, and all the other bacteria did not show any difference (OR 0.86). Finally, serious adverse events were relatively uncommon and more frequent in subjects treated with high-risk antibiotics and in those receiving multiple antibiotic therapy. However, they were never considered attributable to probiotics and were equally distributed between subjects with and without probiotics in the unadjusted analyses (OR 1.06, 95% CI, 0.89–1.26) and adjusted analyses (OR 1.06, 95% CI, 0.89–1.28). 

## 5. Fecal Microbiota Transplantation

As development of CDI, particularly recurrent cases, has been associated with significant variations of gut microbiota composition favoring CD colonization, restoring normal microbiota by fecal transplantations (FMT) from a healthy donor has been considered as a logical and practical solution to reduce the risk of new CDI episodes for many years [84]. However, although this measure has been endorsed by several organizations worldwide [85,86,87] and several reports seem to indicate that it can be effective in patients that were previously treated with standard therapeutic options with poor or no benefit [88,89,90,91,92,93,94,95,96,97], FMT remains debated [98]. Preferred method for administration, optimal donor selection, FMT preparation, route, timing, and number of administrations have not been defined and some of the randomized controlled trials that have studied its efficacy in clinical practice have significant limitations that make results debatable. In addition, it is not established whether FMT is per se effective or can be mainly considered an adjunctive treatment to antibiotics. Finally, safety can be debated as FMT has been associated with the development of severe infection. FDA has recently reported infections caused by enteropathogenic *Escherichia coli* and Shigatoxin-producing *Escherichia coli* that have occurred following use of FMT [99]. Starting from these premises, most of the experts consider FMT a viable treatment option for patients with multiple recurrences that have received without advantage appropriate antimicrobial therapy with metronidazole, vancomycin, or fidaxomicin. On the contrary, FMT cannot be considered a first choice prophylactic treatment to prevent CDI recurrences. 

## 6. Active and Passive Immunization

Clinical manifestations of CDI are due to bacterial toxins. Most of CD strains produce two members of the large clostridial cytotoxin family, toxin A and toxin B, which are encoded by the genes *tcdA* and *tcdB*, respectively, and are located within a five-gene locus known as the PaLoc. A restricted number of CD strains produce a third toxin named CD transferase [100]. As high levels of antibodies against CD toxins A and B correlate with the prevention of primary CDI and low rates of recurrences [101,102,103,104], and the administration of monoclonal antibodies against toxins is effective in reducing CDI recurrences [105], it was thought that active or passive immunization against toxins A and B could reduce the risk of CDI development. Consequently, vaccines and monoclonal antibodies against toxins have been studied. Moreover, the administration of hyperimmune colostrum-containing antibodies against CD was suggested [106]. However, as immunization against CD cannot reduce the risk of colonization, outgrowth, sporulation, and the release of the spores into the environment, favoring increased transmission of CDI, other preventive measures targeting CD surface cellular components or CD spores were developed.

### 6.1. Vaccines

Vaccines against CD are mainly intended for geriatric populations and adults at risk of CDI because of receiving frequent antibiotic treatment or with previous history of CDI. Among those based on CD toxins, a formalin-inactivated preparation of the toxins has been found safe and immunogenic in phase II clinical trials [107,108]. Despite these promising results, the vaccine was retired by the manufacturer after a phase III study showed that the probability of demonstrating that the vaccine could be effective and safe in the prevention of primary CDI was lower than expected [93].

The most advanced toxin-based CD vaccine is a recombinant preparation that contains full-length genetically and chemically inactivated toxins A and B [109,110,111]. Phase I [110] and phase II studies [96] have shown that this vaccine was safe and immunogenic in adults aged 65–85 years [95]. This has supported the implementation of a phase III, placebo-controlled, randomized, observer-blinded study to evaluate the efficacy, safety, and tolerability of the vaccine in adults 50 years of age and older (NCT03090191). Enrolled patients had to receive three doses of the vaccine or placebo and had to be followed for up to 3 years after vaccination. The study is active but is no longer recruiting, with a completion date scheduled for September 2020. No results have been reported to date.

Additionally, worthy of interest is a vaccine based on a recombinant fusion protein comprising relevant epitopes of CD toxins. Even in this case, a phase I study has shown good safety and tolerability and excellent immunogenicity in healthy subjects aged ≥18 years. Moreover, a phase II trial (NCT02316470) in which three doses of the vaccine were given to healthy subjects aged 50 to > 65 showed that seroconversion was achieved in up to 83% of participants for antibodies against both toxins and up to 97% against toxin A alone. Unfortunately, as of now, no phase III trial has been planned, as the manufacturing company is seeking a partner to perform it.

Vaccines based on components of CD spores are in the very initial stage of development. Starting from the evidence that the inactivation of collagen-like exosporium protein BclA1 reduces the ability of CD to colonize mice [112], a vaccine containing BclA1 was prepared and tested in experimental animals [113]. The results were disappointing, as all the animals that had received the vaccine intraperitoneally died after challenge. However, it was suggested that these negative results did not exclude the possibility that a vaccine including collagen-like exosporium proteins may be effective. In the study by Ghose et al. [114], the vaccine was injected intraperitoneally, and this limited immune response as no mucosal immunity, of great importance for intestinal protection, could be evoked. Moreover, the CD strain used for the study encoded a truncated version of BclA1, and the immune response could be lower than desired. As most of the clinically relevant CD strains express full-length BclA proteins, it was thought that a vaccine containing all these proteins, intramuscularly injected, could induce broader and protective immunity.

Another CD vaccine candidate based on structural components of the exosporium layer of pathogen spores is the CdeM vaccine. CdeM is a protein that controls the assembly of the exosporium layer [98], and a vaccine containing this protein was found to protect animals exposed to a virulent CD strain with a dose-dependent effect [113]. The protection of experimental animals from CDI was also reported when a vaccine based on CdeC protein, a second cysteine-rich protein that is supposed to form a protective structure around the spore coat [114], was used [115]. Finally, another vaccine target being considered is CotA, another protein needed for the assembly of CD spores that is able to evoke a strong immune response [116,117,118,119]. The protection of 60% of mice immunized with a vaccine containing CotA was evidenced, suggesting potential possibilities for further development [117].

### 6.2. Passive Immunization

The administration of monoclonal antibodies against CD toxins A and B has been considered a potential measure to prevent CDI recurrences. Two different preparations, supposed to directly neutralize toxin A (actoxumab) and toxin B (bezlotoxumab), respectively, have been developed. Combined intravenous administration of these vaccines showed significant efficacy in an established hamster model of CDI [102]. Moreover, when passive immunization was added to metronidazole or vancomycin in patients with symptomatic CDI, recurrence rates were reduced, although the differences compared to controls were not statistically significant (8% vs. 32%, *p* = 0.06). The difference in recurrence rate reached statistical significance when patients with more than one previous episode of CDI were separately considered (7% vs. 38%, *p* = 0.006) [87]. However, when antibodies were given separately, it was found that only bezlotoxumab could protect experimental animals. Piglets receiving anti-toxin B antibodies were protected in 100% of the cases from the development of systemic CDI with marginal gastrointestinal alterations. In contrast, animals receiving anti-toxin A antibodies suffered from severe systemic and gastrointestinal manifestations with death in the majority of cases [103]. As poor efficacy of actoxumab was confirmed in phase II and III clinical trials in humans [88,104], further development was limited to bezlotoxumab. Data collected from two studies [105] enrolling patients with CDI treated with conventional antibiotic therapy showed that the addition of bezlotoxumab was significantly effective in reducing the risk of recurrence. In the first study, 17% of treated patients had a new episode of diarrhea in the 12 weeks after treatment compared with 28% of patients given placebo. In the second study, new diarrhea episodes occurred in 16% of treated patients and in 26% of placebo patients. In both cases, risk reduction was greater in patients at higher risk of recurrence. Unfortunately, no effect was found on the rate of initial cure. Both bezlotoxumab-treated patients and controls healed in 80% of the cases. This explains why, with the name Zinplava, bezlotoxumab has been approved in both the USA [120] and the European Union [121] only for prevention of recurrences in adults at high risk for recurrent CDI. The antibody was found to be generally safe and well tolerated, as the incidence rate of acute adverse events was similar in treated patients and controls. However, some concerns remain regarding potential health toxicity, as the incidence of heart failure in patients with a history of underlying congestive heart failure was significantly higher among those receiving bezlotoxumab than in controls (12.7% vs. 4.8%). Moreover, more deaths occurred among cases than among controls. Although heart failure and deaths were not infusion-related but were evidenced throughout the 3-month follow-up period and could not be directly linked to bezlotoxumab administration, this problem was considered when bezlotoxumab was licensed for clinical use in the USA. In the insert of the product marketed in the USA, the potential association with heart failure is mentioned, and it is highlighted that this antibody should be prescribed when the supposed benefit outweighs risk [122]. However, this is not reported in the insert of the product marketed in the EU [123].

A particular aspect of passive immunization is oral administration of hyperimmune whey derived from colostrum or mature milk of cows immunized to obtain secretory immunoglobulin A against the two toxins, TcdA and TcdB, as well as CD. Studies carried out in experimental animals are promising as whey protected animals from death, in contrast to standard antibiotic therapy [124]. However, data collected in humans are very few, although in one of these studies, hyperimmune whey was found to be as effective as metronidazole in the prevention of CDI recurrences [125,126,127,128]. Moreover, better characterization of this kind of immunotherapy and more knowledge on its mechanism of action are needed before this prophylactic measure can be considered for clinical use.

## 7. Conclusions

CDI is a significant clinical, social, and economic problem. Table 1 summarizes preventive strategies against CDI. The prevention of primary infection, the diffusion of pathogens, and the development of recurrences seems essential to reduce the total burden due to this pathogen. Unfortunately, despite several studies, the prevention of CD-related problems remains difficult. This seems particularly true for cases that develop in the community and in older children and young adults, as almost all the data presently available have been collected in the hospital and in the elderly population. Practically, only interventions usually recommended for infection control and prevention can reduce CDI, although their use to reduce CD-related problems is not precisely defined, and some aspects remain debated. Moreover, compliance with recommendations is generally poor. Novel strategies for prevention, such as those based on the use of probiotics and active and passive immunization, have not yet been fully developed. Studies specifically devoted to establishing whether probiotics can be effective and defining which type of bacteria, dosage, and duration of administration can reduce the risk of CDI are urgently needed before probiotics can be included among measures for prevention. Totally imaginative and hardly applicative in clinical practice is the use of whey proteins derived from colostrum or breast milk from immunized cows. Several vaccines have been developed. The most advanced methods have significant limitations as they cannot prevent colonization and development of primary CDI. Only vaccines able to face these problems can allow a resolutive approach to the total burden due to this pathogen. 

## Figures and Tables

**Table 1:** Strategies for prevention against *Clostridium difficile* infection (CDI).

Strategy	Action
Infection control measures	Prompt identification of patients with suspected CDI Isolation of suspected and documented cases Disinfection of the patient’s room and objects Use of contact isolation measures such as gloves and gowns
Antibiotic stewardship	Implementation of programmes for the optimization of antibiotic use, is considered one of the most important measures to reduce the selection of host microbiota and the emergence of antimicrobial-resistant bacteria.
Probiotics	Administration of bacteria that confer a health benefit when ingested could reconstitute normal gut microbiota composition and suppress CD colonization in patients given antimicrobial drugs
Active immunization	Toxin-based CD vaccine Vaccine based on a recombinant fusion protein comprising relevant epitopes of CD toxins Vaccine based on components of CD spores Vaccine based on the structural components of the exosporium layer of pathogen spores
Passive immunization	Monoclonal antibodies against CD toxins A (actoxumab) and B (bezlotoxumab) Oral administration of hyperimmune whey derived from colostrum or mature milk of cows immunized to obtain secretory immunoglobulin A against the two toxins, A and B, as well as CD
